# Multiple Reassortment between Pandemic (H1N1) 2009 and Endemic Influenza Viruses in Pigs, United States

**DOI:** 10.3201/1709.110338

**Published:** 2011-09

**Authors:** Mariette F. Ducatez, Ben Hause, Evelyn Stigger-Rosser, Daniel Darnell, Cesar Corzo, Kevin Juleen, Randy Simonson, Christy Brockwell-Staats, Adam Rubrum, David Wang, Ashley Webb, Jeri-Carol Crumpton, James Lowe, Marie Gramer, Richard J. Webby

**Affiliations:** Author affiliations: St. Jude Children’s Research Hospital, Memphis, Tennessee, USA (M.F. Ducatez, E. Stigger-Rosser, D. Darnell, C. Brockwell-Staats, A. Rubrum, D. Wang, A. Webb, J.-C. Crumpton, R.J. Webby);; Newport Laboratories, Worthington, Minnesota, USA (B. Hause, R. Simonson);; University of Minnesota College of Veterinary Medicine, Saint Paul, Minnesota, USA (C. Corzo, K. Juleen, M. Gramer);; and Carthage Veterinary Services, Carthage, Illinois, USA (J. Lowe)

**Keywords:** zoonoses, swine influenza, influenza, endemic, pandemic, reassortant, multiple reassortment, viruses, pigs United States, research

## Abstract

As a result of human-to-pig transmission, pandemic influenza A (H1N1) 2009 virus was detected in pigs soon after it emerged in humans. In the United States, this transmission was quickly followed by multiple reassortment between the pandemic virus and endemic swine viruses. Nine reassortant viruses representing 7 genotypes were detected in commercial pig farms in the United States. Field observations suggested that the newly described reassortant viruses did not differ substantially from pandemic (H1N1) 2009 or endemic strains in their ability to cause disease. Comparable growth properties of reassortant and endemic viruses in vitro supported these observations; similarly, a representative reassortant virus replicated in ferrets to the same extent as did pandemic (H1N1) 2009 and endemic swine virus. These novel reassortant viruses highlight the increasing complexity of influenza viruses within pig populations and the frequency at which viral diversification occurs in this ecologically important viral reservoir.

## Abstract 2

TOC Summary: Viruses belonging to these novel genotypes are indistinguishable phenotypically from endemic swine viruses.

Since its detection in humans in April 2009 (*1*), the pandemic influenza A (H1N1) 2009 virus spread quickly throughout the world. The pandemic virus was first detected in pigs in Canada in early May 2009 (*2*), and at least 14 countries have reported pigs infected with pandemic (H1N1) 2009 viruses (*3*), a few of which have been thoroughly described in the literature: in the Americas during summer 2009 (*4*,*5*); in Norway and Italy during fall 2009 (*6*,*7*); in India in May, June, and November 2009 (*8*); in Hong Kong Special Administrative Region, People’s Republic of China, during October 2009–January 2010 (*9*); and in South Korea and Thailand during December 2009 (*10*,*11*). All of these infections were caused by human-to-pig transmission.

In the United States, H1N1 subtypes of the classical swine influenza lineage (now designated as H1α) dominated from 1918 through 1998. In ≈1998, influenza (H3N2) triple reassortant viruses appeared, accompanied by a transient increase in disease severity (*12*,*13*). These triple reassortants contained polymerase acidic (PA) and polymerase basic 2 (PB2) genes of avian virus origin; hemagglutinin (HA), neuraminidase (NA), and polymerase basic 1 (PB1) genes of human virus origin; and matrix (M), nucleoprotein (NP) and nonstructural (NS) genes of classical swine virus origin (*13*). After these viruses appeared, multiple reassortment occurred that combined different HA and NA genes with the triple reassortant internal gene (TRIG) cassette (PA, PB1, PB2, NP, M, and NS) (*14*–*20*).

In addition to H1α, 3 distinct lineages of H1 hemagglutinin have been defined and characterized: H1β strains, first detected in 2001–2002; H1δ (or “seasonal human-like” swine H1) strains in 2003–2005; and H1γ strains in 1999–2000 (*19*,*21*). Soon after the appearance of pandemic (H1N1) 2009 viruses (whose HA clusters with the swine H1γ viruses) in pigs, the first reassortment event with an endemic swine influenza virus was reported in pigs in Hong Kong. This virus, A/swine/201/2010, contained a Eurasian swine lineage HA, a pandemic (H1N1) 2009 NA, with the TRIG cassette (*9*). Subsequently, a reassortant with 7 pandemic (H1N1) 2009 gene segments and a swine N2 gene was found in Italy (*22*), and a reassortant with 7 pandemic (H1N1) 2009 gene segments and a swine N1 gene was found in Germany (*23*). Considering the known circulation of TRIG-containing endemic and pandemic (H1N1) 2009 viruses in pigs, the chance for similar reassortment to occur in the United States also seemed high.

We describe the isolation of 9 pandemic (H1N1) 2009/endemic swine reassortant influenza viruses representing 7 distinct genotypes in pigs in the United States. Our study highlights the effect of reverse zoonotic transmission of the pandemic virus on this population.

## Materials and Methods

### Samples

Samples used in this study were nasal swabs or lungs collected from pigs with clinical signs of respiratory disease, with the exception of A/swine/Indiana/240218/2010, which was isolated from a healthy pig within the framework of an active swine influenza surveillance program. In this program, nasal swab specimens had been randomly collected on a monthly basis since June 2009 from commercial farms in Iowa, Indiana, Minnesota, North Carolina, and Illinois. A/swine/Indiana/240218/2010 was 1 of 176 viruses detected. Vaccination status of the pigs was unknown. The specimens were transported in virus transport media at 4°C to the laboratory (Newport Laboratories, Worthington, MN, USA, or St. Jude Children’s Research Hospital, Memphis, TN, USA) for influenza screening. Samples were either tested within 48 h or frozen at −80°C before being processed.

### RNA Extraction and Real-time Reverse Transcription PCR

RNA was extracted either with a MagMAX−96 AI/ND viral RNA isolation kit (Applied Biosystems/Ambion, Austin, TX, USA) on a Kingfisher Flex (Thermo Scientific, Rockford, IL, USA), or with QIAGEN viral RNA kit (QIAGEN, Valencia, CA, USA), following the manufacturers’ instructions. Real-time reverse transcription PCR (rRT-PCR) was performed to initially screen for all influenza A viruses (*24*). Positive samples were then screened specifically for swine H3 and H1 HA genes (*25*) and the pandemic (H1N1) 2009 M gene (*24*). One-step RT-PCR was performed by using the QIAGEN 1-step RT-PCR kit (QIAGEN), 600 nmol/L of each primer, 300 nmol/L of the probe, and 1.4 mmol/L MgCl_2_. The ABI Fast real-time PCR system 7500 thermocycler and corresponding software (Applied Biosystems, Foster City, CA, USA) were used with the following cycling conditions: 50°C for 30 min, 95°C for 15 min, followed by 40 cycles of 95°C for 10 sec, and 60°C for 30 sec. For the growth curves, viral titers were monitored by rRT-PCR by using the method described by Harmon et al. (*26*).

### Virus Isolation and Growth

Swab samples were added to MDCK or swine testicle (ST) cells (American Type Culture Collection) as described (*27*,*28*). Virus isolates were identified by HA assay, as described in the World Health Organization manual on animal influenza diagnosis and surveillance (*28*). Specimens were plaque purified 2× on either MDCK or ST cells. Isolates were then characterized by full genome sequencing. Virus growth characteristics were compared on ST cells. Approximately 1.0 50% tissue culture infectious dose per milliliter of each virus was added to a confluent monolayer of ST cells. An aliquot was immediately removed after inoculation and every 24 hours through 4 days postinoculation. Samples were analyzed by using rRT-PCR that targeted the M gene and by titration on ST cells by using the method of Spearman-Karber.

### Sequencing and Sequences Analysis

Specimens were sequenced by using an Illumina Genome Analyzer (Illumina, Inc., San Diego, CA, USA). An RT-PCR was performed on RNA templates by using Uni-12 and Uni-13 primers to amplify all 8 segments in 1 reaction with Invitrogen SuperScript III One-Step Reverse Transcriptase and Platinum *Taq* HiFi (Invitrogen, Carlsbad, CA, USA). Polymerase gene primers were added to optimize the sequencing reaction (*29*). The obtained double-stranded DNA was sonicated in a Covaris AFA (Covaris, Woburn, MA, USA) until a broad peak at 200 bp appeared. The 3′ overhangs were removed from the sheared DNA by end repair, a Poly-A tail was added, and adapters were then ligated to the DNA fragments by using New England Biolabs (NEB) kits E6050L, E6053L, and E6056L (NEB, Ipswich, MA, USA). The ligation products were purified by gel electrophoresis by using E-Gel SizeSelect 2% agarose precast gels (Invitrogen). Index sequences were added to the DNA samples by Phusion DNA polymerase (NEB) before they were loaded on the illumina sequencer.

For sequence analyses, samples were de-multiplexed and each genome was assembled by using CLC Genomics Workbench software (CLC bio, Germantown, MD, USA) by running a high stringency de novo assembly. Sequences were compared by using BioEdit (*30*) and ClustalW (*31*). Phylogenetic analyses were performed by using MEGA version 4.0.2 (*32*). The sequences of the 9 influenza viruses we studied were submitted to GenBank under accession nos. CY086877–CY086942.

### Assessment of Virus Pathogenicity in Ferrets

The pathogenicity of selected influenza viruses was tested in male ferrets (*Mustela putorius furo*) 3–4 months of age obtained from Triple F Farms (Sayre, PA, USA). All ferrets were seronegative for seasonal influenza A (H1N1) and (H3N2) viruses, pandemic (H1N1) 2009, and influenza B viruses by hemagglutination-inhibition assay. Five ferrets were inoculated intranasally under light isoflurane anesthesia with 10^6^ 50% egg infectious dose (EID_50_) of the subtype H3N2 reassortant, A/swine/Minnesota/239105/2009, in 1 mL of sterile phosphate-buffered saline. Pandemic (H1N1) 2009 strain A/Tennessee/560–1/2009 (TN/560/09) and influenza (H3N2) strain A/swine/Texas/4199–2/98 (sw/TX/4199/98) were used as controls for comparison. Clinical signs of infection, relative inactivity index (*33*), weight, and temperature were recorded throughout the 12-day study period.

To monitor virus shedding, nasal washes were collected from ferrets on days 3, 5, and 7 postinoculation as described (*34*). The virus titers were determined as log_10_ EID_50_/mL. The limit of virus detection was 0.5 log_10_ EID_50_/mL. For calculation of the mean, samples with a virus titer <0.5 log_10_ EID_50_/mL were assigned a value of 1.

All animal experiments were performed in BioSafety level 2+ facilities at St. Jude Children’s Research Hospital (Memphis, TN, USA). All animal studies were approved by the St. Jude Children’s Research Hospital Animal Care and Use Committee and were conducted according to applicable laws and guidelines.

## Results

### Identification of Endemic Swine–Pandemic (H1N1) 2009 Influenza Virus Reassortants

**Figure 1:**
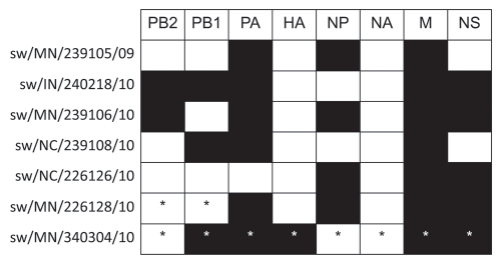
Lineages of North American reassortant swine influenza viruses identified through phylogenetic analyses. Pandemic and endemic gene segments are represented in black and white, respectively. *Denotes partial sequences. Isolates sw/NC/226124/10 and sw/NC/226125/10 (not shown) have the same genotype as sw/NC/226126/10. PB2, polymerase basic 2; PB1, polymerase basic 1; PA, polymerase acidic; HA, hemagglutinin; NP, nucleoprotein; NA, neuraminidase; M, matrix; NS, nonstructural.

**Figure 2:**
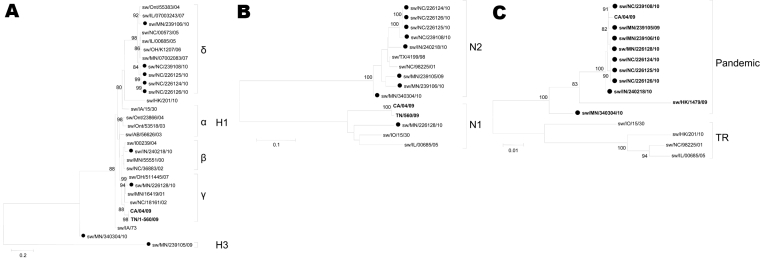
Phylogenetic trees of pandemic reassortant swine influenza viruses compared with currently circulating swine influenza strains: A) hemagglutinin (H); B) neuraminidase (N); C) matrix genes. The trees were constructed by using the neighbor-joining method (Kimura 2-parameter) with 1,000 bootstrap replicates. Only bootstrap values >74 are shown. Swine reassortant strains characterized in this study are indicated with a closed circle. **Boldface** indicates pandemic segments. Greek letters indicate virus genogroups; α represents classical swine influenza virus and δ seasonal human-like swine influenza virus. TR indicates swine triple reassortant influenza virus. Scale bars indicate nucleotide substitutions per site.

During routine surveillance for influenza viruses in pigs, 9 reassortant viruses were detected during 2009 and 2010. These viruses came from asymptomatic animals or from animals showing classic influenza symptoms, including coughing, respiratory distress, fever, or nasal discharge. These viruses were detected in Minnesota, Indiana, and North Carolina. Complete genome sequences were obtained for 7 strains: A/swine/Indiana/240218/2010(H1N2) (sw/IN/240218/10), A/swine/Minnesota/239105/2009(H3N2) (sw/MN/239105/09), A/swine/Minnesota/239106/2010(H1N2) (sw/MN/239106/10), A/swine/North Carolina/239108/2010(H1N2) (sw/NC/239108/10), A/swine/North Carolina/226124/2010(H1N2) (sw/NC/226124/10), A/swine/North Carolina/226125/2010(H1N2) (sw/NC/226125/10), and A/swine/North Carolina/226126/2010(H1N2) (sw/NC/226126/10). Partial sequences were obtained from 2 additional samples, A/swine/Minnesota/340304/2010(H1N2) (sw/MN/340304/10), and A/swine/Minnesota/226128/2010(H1N1) (sw/MN/226128/10). Of the 9 viruses, 8 displayed HA and NA genes of endemic swine influenza viruses and pandemic (H1N1) 2009 M gene segments; the origin of the remaining gene segments differed depending on the virus (Figure 1). The phylogeny of HA, NA, and M genes were compared with reference strains (Figure 2, panels A, B, C, respectively). Only sw/NC/226124/10, sw/NC/226125/10, and sw/NC/226126/10 had identical genotypes with pandemic (H1N1) 2009 NP, M, and NS genes. These 3 viruses were isolated during a short period from the same general location: sw/NC/226124/10 and sw/NC/226125/10 came from the same farm, and sw/NC/226126/10 was isolated 48 hours later at a neighboring farm.

Eight of the 9 reassortant viruses were successfully isolated on either MDCK or ST cells. Sw/MN/340304/10 could not be isolated, most likely because of lack of initial material, because this specimen had the highest cycle threshold value by rRT-PCR targeting the M gene (cycle threshold value 36). Isolates were plaque-purified on MDCK or ST cells, and the genotypes were all confirmed. Taken together these data show that several novel genotypes of swine influenza viruses had been generated after the reverse zoonotic transmission of pandemic (H1N1) 2009 virus to pigs.

### Replication and Pathogenicity of Pandemic Reassortant Influenza Virus In Vitro and In Vivo

**Figure 3:**
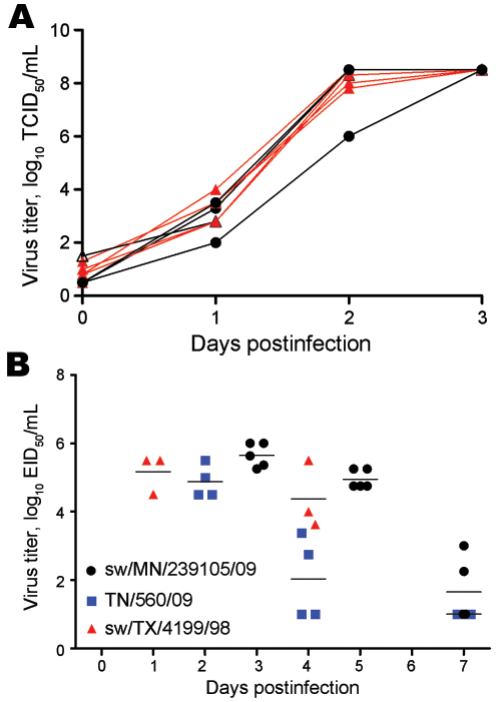
Replication of North American reassortant and endemic swine influenza viruses on swine testicle (ST) cells and in ferrets. A) The growth of 6 endemic swine viruses and 3 reassortant viruses (sw/MN/239105/09, sw/MN/239106/10, and sw/NC/239108/10) from 2009–2010 were analyzed in vitro. One curve corresponds to 1 isolate. Black lines and symbols indicate swine pandemic influenza reassortant viruses; red lines and symbols indicate swine triple reassortant (TR) influenza viruses. The progeny viruses released from infected ST cultures were collected at the indicated time points and titrated in ST cells by performing a 50% tissue culture infectious dose (TCID_50_) assay. Negative TCID_50_ titers were given the value 0.5; log_10_ TCID_50_ >8.5 were given the value 8.5. B) Virus titers in the upper respiratory tract (nasal washes) of ferrets infected with 10^6^ 50% egg infectious dose (EID_50_) pandemic (H1N1) 2009 TN/560/09 (*35*); 10^6^ TCID_50_ swine TR influenza virus sw/TX/4199/98; or 10^6^ EID_50_ swine pandemic reassortant virus sw/MN/239105/09. Values are the mean ± SD for 4, 3, and 5 ferrets for TN/560/09, sw/TX/4199/98, and sw/MN/239105/09, respectively.

To understand whether the increased genetic diversity created through the reassortment was associated with an increase in phenotypic diversity, select reassortants were assessed for growth in vitro and in ferrets. The growth characteristics of 6 endemic viruses from 2009–2010 and 3 pandemic reassortant viruses (sw/MN/239105/09, sw/MN/239106/10, and sw/NC/239108/10) were compared on ST cells. We found no difference in replication potential between any of these viruses, which suggests that no selective growth advantages had occurred through reassortment (Figure 3, panel A).

Because of the recent zoonotic transmissions of triple reassortant swine influenza (H3N2) viruses in the United States during 2010 (*36*), we also sought to determine whether reassortment had the potential to lead to a more pathogenic virus than previously circulating swine influenza strains. We used the ferret model to assess this possibility. Each of 5 ferrets was inoculated with 10^6^ EID_50_ of sw/MN/239105/09 (H3N2). This reassortant virus caused only mild clinical signs (relative inactivity index ≈0.1) without marked weight changes or body temperature elevation (maximum of 3% weight loss, and 0.4°C increase in temperature; data not shown). The virus replicated to similar titers, as did the pandemic (H1N1) 2009 virus, TN/560/09, and the triple reassortant swine subtype H3N2 virus, sw/TX/4199/98, with a peak titer of infection of 5–6 log_10_ of virus and with viral clearance occurring ≈1 week postinfection (Figure 3, panel B). The similar disease and growth property of these viruses in ferrets again suggested that no unusual biologic properties had been inherited upon reassortment.

## Discussion

During 1998–2009, reassortment of influenza viruses in US pigs occurred relatively frequently (*37*). The genotypes of the viruses generated through these reassortments typically contained different swine or human influenza virus HA and NA genes in combination with the TRIG cassette (*13*–*20*). These reassortant viruses provided 6 of the 8 gene segments to the pandemic (H1N1) 2009 virus (*38*). Thus, and as indicated by similar events in other geographic locations, it was not unexpected that reassortment between pandemic (H1N1) 2009 and endemic swine influenza viruses would occur in US pigs after identification of the former virus in this population.

Somewhat unpredicted, however, was the number of reassortants that we identified in this study; 7 distinct viral genotypes were characterized. Although different genotypes were detected, each had an M gene of pandemic (H1N1) 2009 origin, a novel gene segment introduced into this animal population after human-to-pig transmission of the pandemic strain. The TRIG cassette in the reassortant viruses, a cassette that had remained relatively unchanged since 1998, was disrupted to include not only the M gene segment but also variably the NS, NP, and PA genes of pandemic (H1N1) 2009 virus. Because the pandemic virus contains M and NA gene segments from Eurasian-lineage swine influenza viruses and PB2, PB1, PA, NP, and NS gene segments from TRIG viruses, it is not surprising that several pandemic (H1N1) 2009 genes could be introduced into endemic US swine influenza viruses without altering the viability of the progeny viruses.

The inclusion of the pandemic (H1N1) 2009 M gene in the reassortants suggests a selective advantage to viruses containing it, although we were unable to measure any phenotypic differences in these viruses in our in vitro and in vivo assays. One phenotype that we did not measure was transmission, and it is tempting to speculate that the pandemic (H1N1) 2009 M gene segment could play a role here, both in terms of its selection in the reassortants described and in the human pandemic virus itself. Further studies are required to test this hypothesis.

The effect of these reassortants on the US pig industry is somewhat difficult to predict, although on the basis of the data generated here it is not likely to be great in terms of animal health. Antigenically, the reassortant influenza viruses carried HA genes already within the population (either endemic or pandemic (H1N1) 2009 viruses), and we were unable to detect any replication differences or unusual clinical signs in the field. Thus, and because HA-specific immunity is the target of current vaccines, the generation of these viruses is not expected to have any adverse effect on vaccine efficacy levels or disease severity in the field unless further adaptive changes occur as a result of continued circulation of these viruses.

Although the reassortants invariably contained the pandemic virus M gene and, with the exception of 1 reassortant, endemic virus HA and NA genes, the fact that we only saw viruses of the exact same genotype in limited spatial and temporal space suggests that there is no single dominant reassortant yet. Indeed, it is possible that these reassortants are generated but quickly displaced by other influenza viruses. Nevertheless, the data presented here once again highlight the dynamic nature of influenza viruses in pig populations and the continued monitoring of viruses in US pigs at the level of full genome sequencing is absolutely required.
